# Analyzing false‐negative results detected in low‐risk non‐invasive prenatal screening cases

**DOI:** 10.1002/mgg3.1185

**Published:** 2020-02-18

**Authors:** Ying Lin, Dong Liang, Yan Wang, Hang Li, An Liu, Ping Hu, Zhengfeng Xu

**Affiliations:** ^1^ Department of Prenatal Diagnosis Women’s Hospital of Nanjing Medical University Nanjing Maternity and Child Health Care Hospital Nanjing Jiangsu Province China

**Keywords:** common trisomy, false‐negative results, microdeletion/microduplication, non‐invasive prenatal screening, partial aneuploidy

## Abstract

**Background:**

The non‐invasive prenatal screening (NIPS) has been introduced into clinical practice with a high sensitivity and specificity. Although the false‐negative results are inevitable and important, limited false‐negative NIPS results have been reported and studied previously. In this study, we aim to report and analyze false‐negative results detected in the NIPS cases with a low‐risk result.

**Methods:**

NIPS was performed using whole‐genome massively parallel shotgun sequencing for screening common trisomies, rare autosomal aneuploidies, and subchromosome copy number variants. All the NIPS cases with a low‐risk result performed in our center in 2017 were followed‐up using medical records and telephone interview at 3 months after delivery. Fetal ultrasound results and available genetic diagnostic testing results were collected for pregnancies with adverse outcomes. The genetic diagnostic testing referred to chromosomal microarray analysis or fluorescent in situ hybridization on amniotic fluid cells, fetal skin tissue, neonatal peripheral blood, or available placental biopsies.

**Results:**

By following‐up 10,975 low‐risk results, we found 166 NIPS cases with adverse pregnancy outcomes, in which eight cases had diagnostic testing. Among them, four false‐negative cases were confirmed, including one trisomy 18 caused by placental mosaicism, one mosaic tetrasomy 12p, and 2 microdeletion/microduplication cases.

**Conclusion:**

Our results revealed that mosaicism contributes to a major cause of false negative in NIPS, and highlighted the importance of ultrasound in identifying these false‐negative results.

## Graphical abstract

In this study, we performed a comprehensive follow‐up on a cohort consisting 10975 NIPS cases with a low‐risk result from a single center, which revealed four false‐negative cases. Our study provides information regarding the etiologies of false‐negative results, which will contribute to improve both the NIPS technique and the related perinatal care in clinic.

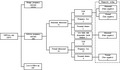

## 1 INTRODUCTION

Cell‐free fetal DNA (cfDNA)‐based non‐invasive prenatal screening (NIPS) has been routinely applied for evaluating the risks of fetal trisomies 21, 18, and 13 (Fiorentino et al., 2017; Liang et al., 2018; Zhang et al., 2015). Other chromosomal imbalances, such as sex chromosomal aneuploidies, copy number variations (CNV), and rare autosomal trisomies (RAT), have also been included into the expanded detection scope, although its regular application in clinic is still controversial (Benn & Grati, 2018; Chitty, Hudgins, & Norton, 2018; Schwartz, Kohan, Pasion, Papenhausen, & Platt, 2018). However, as a screening method, false positive and false negative of NIPS results were inevitable (Cheung et al., 2018; Hochstenbach et al., 2015; Mennuti, Cherry, Morrissette, & Dugoff, 2013; Pan et al., 2013). As recommended by major guidelines, pregnant women with a high‐risk NIPS result should be counseled and offered diagnostic testing, which minimizes the effect of false‐positive results. On the other hand, for pregnancies with a low‐risk NIPS result, invasive prenatal diagnosis is not recommended unless abnormal findings associated with chromosomal abnormalities are detected during the subsequent examinations. The birth of children with chromosomal anomalies missed by NIPS will bring huge stress for the families, both financially and emotionally. Therefore, false‐negative results lead to a worse pregnancy consequence than false‐positive results.

It is important to understand the biology behind these false‐negative cases, which will improve genetic counseling and prenatal management in clinic. However, due to the limited number of false‐negative NIPS cases been studied and reported (Hartwig, Ambye, Sorensen, & Jorgensen, 2017), the underlying etiology has not been clarified. In this study, we performed a comprehensive follow‐up on a cohort consisting 10,975 NIPS cases with low‐risk results from a single center, which revealed four false‐negative cases, including one with trisomy 18, one with tetrasomy 12p, and two with microdeletions/microduplications.

## 2 MATERIALS AND METHODS

### 2.1 Study cohort

This study was approved by the institutional review board of the Nanjing Maternity and Child Health Care Hospital. From January 2017 to December 2017, 11,175 NIPS cases were performed at our center of prenatal diagnosis in Nanjing Maternity and Child Health Care Hospital. Among them, 10,975 cases with a low‐risk NIPS result were included in this study. Informed consent was signed at pre‐test counseling in all the cases.

### 2.2 Non‐invasive prenatal screening

Five milliliters peripheral blood of pregnant women were collected using EDTA anticoagulant tube and centrifuged within 8 hr to extract the plasma. CfDNA purified from the plasma using the fetal chromosome aneuploidy test kit developed by BGI (BGI, China). After library construction, all libraries were sequenced on the BGI‐500 platform (BGI, China). Sequencing reads of 35 bases were trimmed and aligned to a universal unique read set, incised from the human reference genome (hg19, NCBI build 37). Quality control criteria included minimum unique read number of 3.5 Mb, GC content range of 38%–42%, and minimum fetal fraction of 3.5%. Analysis was performed for all samples on aneuploidies of chromosomes 13, 18, 21, X, and Y, as well as other genome‐wide RAT and subchromosome CNV (Chen et al., 2013; Jiang et al., 2012).

### 2.3 Clinical follow‐up on the low‐risk nips results

Pregnancies with low‐risk NIPS results were recommended for routine prenatal care and interviewed by telephone at 3 months after delivery according to the guideline published by Chinese government, as reported previously (Liang et al., 2018). Information, including pregnancy outcomes, date of birth, sex, newborn physical examination results, and maternal physical, were recorded. Fetal ultrasound results and available neonatal/fetal genetic diagnostic testing results were collected for pregnancies with adverse outcomes.

### 2.4 Chromosomal microarray analysis (CMA)

Genomic DNA were extracted using QIA amp DNA Mini Kit (Qiagen). In our center, tissue samples, such as placental tissues and fetal tissues, were tested using the platform of human cyto12 SNP array, whereas other samples, such as peripheral blood samples and amniotic fluid cells, were tested using the platform of CytoScan 750K array. For Illumina array platform, Human cyto12 SNP array (Illumina) comprising approximately 300,000 SNP probes was applied for the whole‐genome scan. Molecular karyotype analysis was performed by KaryoStudio V 1.4.3.0 (Illumina). For Affymetrix array platform, CytoScan 750K array (Affymetrix) comprising approximately 550,000 copy number CNVs probes and 200,000 SNP probes were applied for the whole‐genome scan. Molecular karyotype analysis was performed by Affymetrix Chromosome Analysis Suite (ChAS). CNVs of two array platforms were all called at an effective minimal resolution of 100kb involving at least 10 contiguous probes.

### 2.5 Fluorescent in situ hybridization (FISH) analysis

FISH analysis was performed according to the manufacturer's protocols (VYSIS Inc.) using commercially available subtelomeric specific probes.

## 3 RESULTS

**Figure 1:**
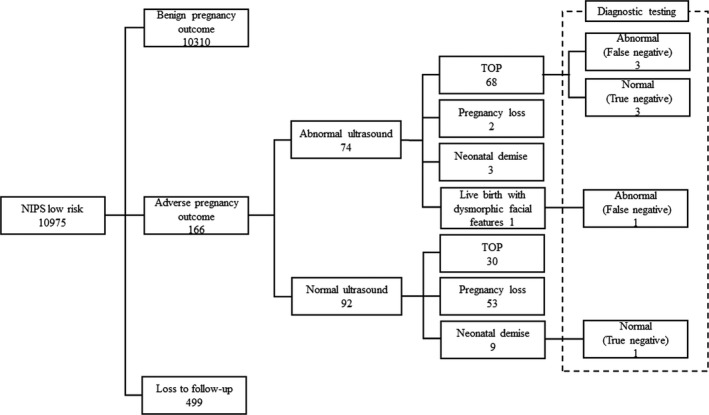
Pregnancy outcomes of the 10,975 NIPS cases with a low‐risk result. TOP, termination of pregnancy

A total of 11,175 pregnancies had NIPS in our center in 2017. The average maternal age was 34.5 years (ranging from 17 to 47 years), and the average gestation was 17^+6^ weeks (ranging from 12^+0^ to 26^+5^ weeks). In all, 200 (1.79%) high‐risk results and 10,975 (98.21%) low‐risk results were reported. Among the low‐risk results, 10,476 (95.45%) cases were successfully followed‐up, which revealed 10,310 cases with benign pregnancy outcomes, 166 cases with adverse pregnancy outcomes, including neonatal demise, pregnancy loss, termination of pregnancy (TOP), and live birth with dysmorphic facial features, associated with or without fetal ultrasound findings (Figure 1).

**Figure 2:**
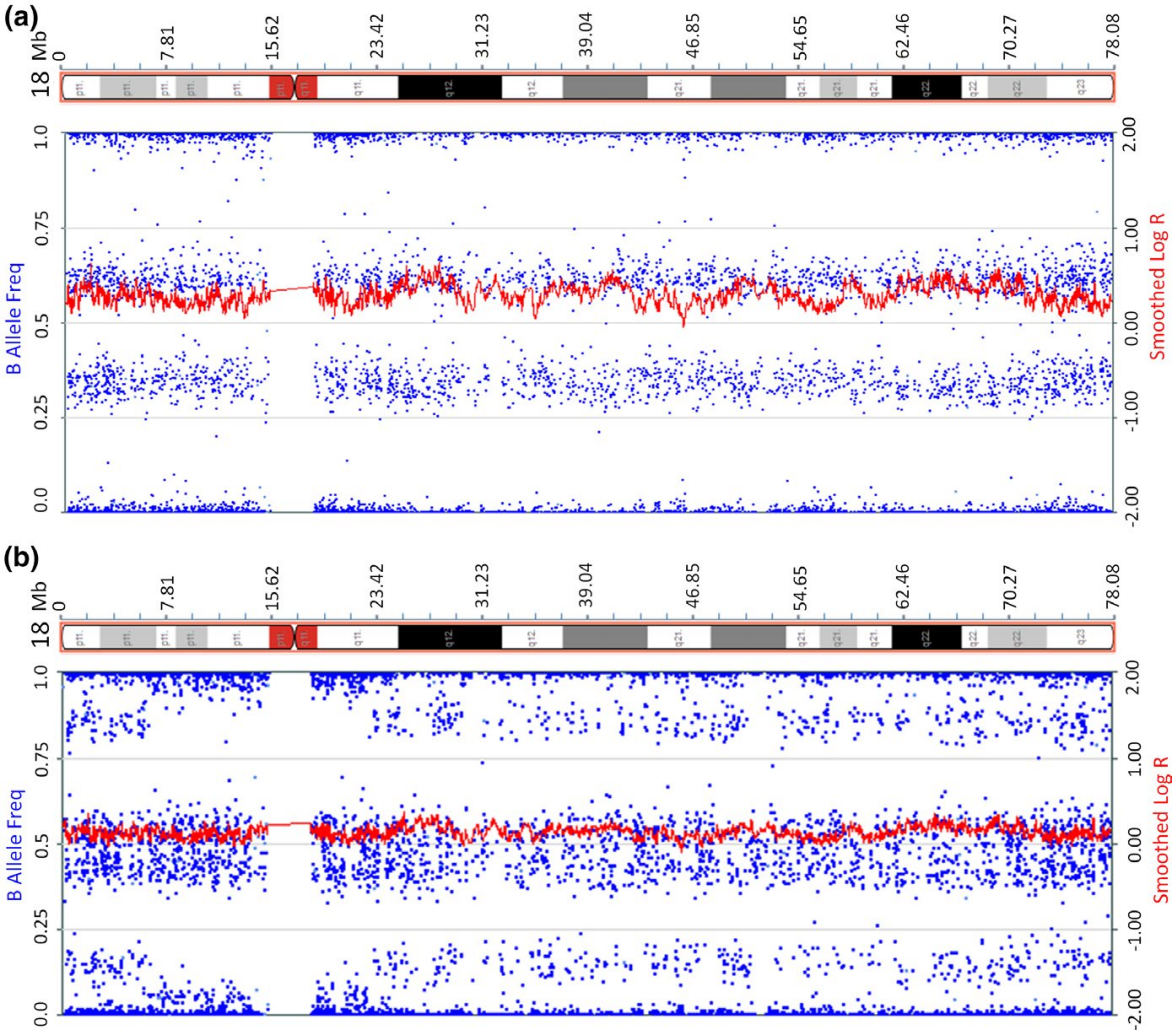
The CMA results for the false negative of trisomy 18. (a) CMA result using the fetal skin tissue revealed a trisomy 18 karyotype; (b) CMA result using the maternal surface of placenta tissue revealed a mosaic karyotype, with the trisomy 18 mosaicism at about 10%–20%

**Figure 3:**
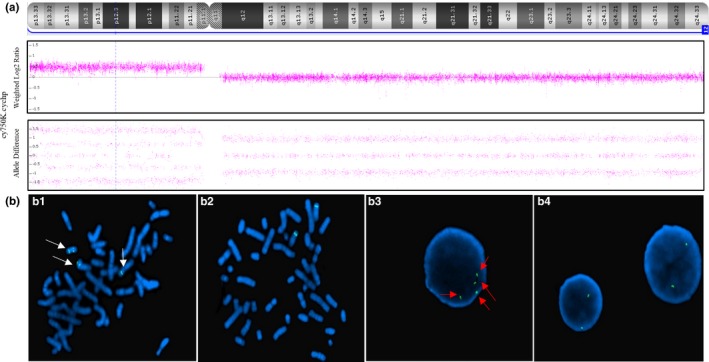
Diagnostic test result for the false‐negative case of mosaic tetrasomy 12p. (a) CMA result revealed a 34.6 Mb duplication at 12p13.33p11.1; (b) Representing images in FISH results. The 12p telomere is labeled in green. (b1) Metaphase spread showing the additional signal at the end of 12pter, an event seen in only 3 of 30 metaphases; (b2) Metaphase spread showing the normal signal; (b3) Interphase nuclei showing a 4‐copy 12pter signal, an event seen in 8 of 50 interphase nuclei. Noting that the interphase result is consistent with tetrasomy 12p mosaicism. (b4) Interphase nuclei showing the normal signal. CMA, chromosomal microarray analysis. FISH, fluorescent in situ hybridization

**Figure 4:**
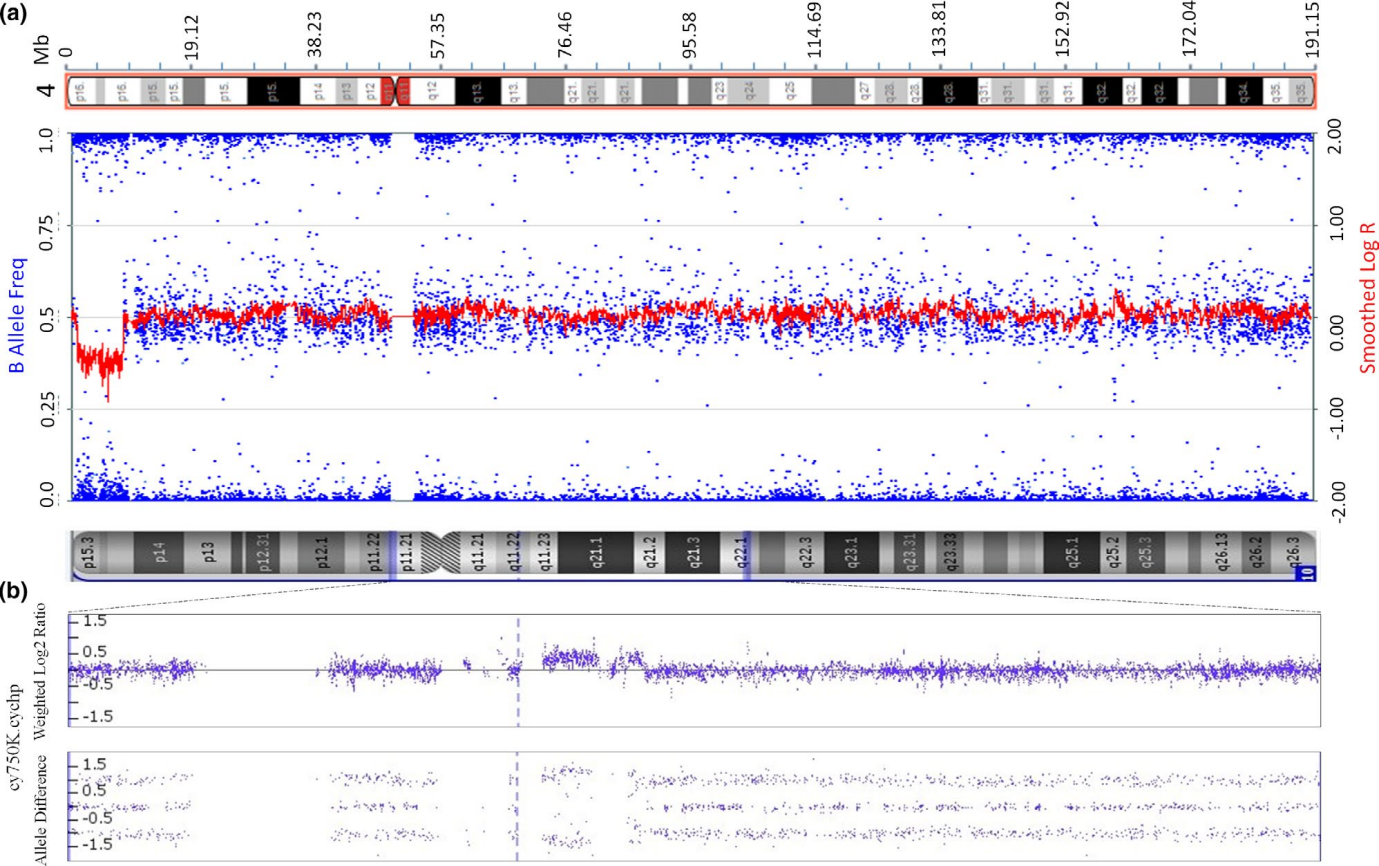
The confirmatory CMA test results for the two false‐negative cases with microdeletion/microduplication. (a) The CMA result showed a 7.07 Mb deletion at the region of 4p16.3‐p16.1. (b) The CMA result showed two duplications at the region of 10q11.22‐q11.23 and 10q11.23 with size of 2.41 Mb and 837 kb. CMA, chromosomal microarray analysis

Eight of the 166 cases with adverse pregnancy outcomes had diagnostic testing for the fetuses, in which four false‐negative results were confirmed, including one aneuploidy (trisomy 18), one partial aneuploidy (tetrasomy 12p), and 2 subchromosomal microdeletion/microduplication cases. The clinical information of the four false‐negative cases, including NIPS results, ultrasound findings, and pregnancy outcomes, are summarized in Table 1. The confirmatory genetic testing for the fetuses varied among these cases. For the case of trisomy 18, placental biopsies, as well as fetal skin tissues, were retrieved and sent for CMA. The result on the fetal skin tissue revealed trisomy 18. However, results on the placenta revealed a mosaicism consisting low level of trisomy 18 cells (Figure 2), indicating mosaicism is the main cause in this case. For the case of mosaic tetrasomy 12p, a boy with dysmorphic facial features was born, including high arched palate, low‐set ears, and claw hands. Peripheral blood sample from the neonate was collected for genetic analysis using CMA and FISH. CMA result revealed a 34.6 Mb duplication involving the entire short arm of chromosome 12, and FISH result confirmed isochromosome of 12p in 3 of 30 metaphases [ish i(12)(p10)(RP11‐55I13++)[3/30]] and in 8 of 50 interphases [nuc ish(RP11‐55I13 × 4)[8/50]](Figure 3), considered as tetrasomy 12p mosaicism or Pallister‐Killian Syndrome (PKS). For the case with microdeletion, the pregnancy was terminated after the abnormal ultrasound result was obtained, and the fetal skin tissue was sent for CMA, which showed a 7.07 Mb deletion on the short arm of chromosome 4, known as Wolf‐Hirschhorn syndrome. As for the case with microduplication, amniocentesis was performed before termination, and the result revealed two small pathogenic duplications (2.4 Mb and 837 kb) on the long arm of chromosome 10 (Figure 4).

**Table 1:** Demographic characteristics and clinical information associated with the four false‐negative cases

Case	Maternal age (years)	GA at NIPS blood drawn (weeks)	Fetal fraction (%)	GA at ultrasound examination (weeks)	Abnormal ultrasound findings	Sample type	Outcome	CMA results	FISH results
1	26	17^+3^	7.9%	18^+3^	Spina bifida, VSD, bilateral cleft lip, bilateral choroid plexus cysts, bilateral rocker bottom feet, lemon‐shaped head	Fetal skin	TOP	arr[hg19] (18) × 3	NA
						Placental		arr[hg19] (18) × 2 ~ 3	
2	28	20^+2^	11.11%	23^+0^	Bilateral renal pelvis, hydramnios	Neonatal PB	Live birth with dysmorphic facial features, including high arched palate, low‐set ears, claw hands	arr[hg19] 12p13.33p11.1(173,786–34,835,641) × 2 ~ 4, 34.6 Mb	ish i(12)(p10)(RP11−55I13++)[3/30] nuc ish(RP11−55I13 × 4)[8/50]
3	30	20^+0^	11.36%	26^+3^	VSD, persistent left superior vena cava, single umbilical artery, absent nasal bone, cerebellar vermis hypoplasia	Fetal skin	TOP	arr[hg19] 4p16.3p16.1(848,280–7,922,502) × 1, 7.07 Mb	NA
4	33	16^+6^	NA	25^+4^	VSD	Amniotic fluid	TOP	arr[hg19] 10q11.22q11.23(48,698,612–51,109,013) × 3, 2.41Mb, 10q11.23 (51,620,168–52,457,367) × 3, 837kb	NA

Abbreviations: CMA, chromosomal microarray analysis; FISH, fluorescent in situ hybridization; GA: gestational age; NA: not available; NIPS, non‐invasive prenatal screening; PB, peripheral blood; TOP, termination of pregnancy; VSD: ventricular septal defect.

## 4 DISCUSSION

Although NIPS has been applied into clinic with a critical contribution to prenatal care, false negatives are inevitable due to the nature of this cfDNA‐based screening method. Previous studies raised the importance of studying the false‐negative results. However, Hartwig et al. systematically searched and reviewed 22 papers related with discordant NIPS results between 2013 and 2016, and found only 24 false‐negative results for trisomies 21, 18, and 13 were reported, with 45.83% (11/24) had unexplained causes (Hartwig et al., 2017). There are two possible reasons for the limited false‐negative cases reported. First, the frequency of false‐negative results in NIPS is relatively low, reported as about 1 in 10,000 NIPS cases for common trisomies (Liang et al., 2018; Zhang et al., 2015). Second, a comprehensive follow‐up on the NIPS cases, especially for the cases with a low‐risk result, is time‐consuming and difficult to perform in most commercial laboratories. In this way, the frequency of false negatives could be underestimated, and false‐negative results of RAT and microdeletions/microduplications are more rarely reported. In this study, we successfully followed‐up 10,476 cases from 10,975 NIPS cases with a low‐risk result performed in our center in 2017. Confirmatory genetic testing was performed in 8 cases among the 166 pregnancies with adverse outcomes. Notably, half of the eight cases had abnormal chromosome results which were missed by NIPS, including one case of trisomy 18, one case of tetrasomy 12p, one microdeletion case, and one microduplication case. Our results showed the importance of performing genetic diagnosis for low‐risk results with adverse pregnancy outcomes in NIPS.

Due to the fact that the circulating fetal cfDNA mainly derives from the placenta, an inaccurate negative result will be generated if there is insufficient amount of cfDNA derived from abnormal genome in the maternal plasma caused by mosaicism in the placenta, even if the fetal karyotype contains aneuploidy or CNV. Gao et al. reported a condition of 20%–30% mosaicism of placental cells with karyotype 48, XXX, +18 that led to a partially inaccurate NIPS result (Gao, Stejskal, Jiang, & Wang, 2014). They believed that the 7.4% fetal cfDNA fraction and the 30% trisomy 18 mosaicism resulted in a reduced (<2.2%) effective fetal fraction for trisomy 18 detection. In our case of false‐negative trisomy 18, the general fetal fraction was about 8%, and test result from placental DNA revealed a low‐level trisomy 18 mosaicism (10%–20%) which resulted in a lower fetal fraction of trisomy 18 (0.8%–1.6%). Given the cfDNA containing trisomy 18 component was lower than 2%, the fetal aneuploidy would be missed by most NIPS assays. Similarly, in our case of false‐negative tetrasomy 12p, the FISH result from neonatal peripheral blood revealed a low‐level mosaicism of tetrasomy 12p (10%–20%). Assuming the mosaicism level was similar in the placenta, the cfDNA fraction of tetrasomy 12p was lower than 2.2%, which was theoretically undetectable using NIPS.

Although several previous work indicated NIPS is capable of detecting large chromosomal imbalances (Lefkowitz et al., 2016; Wapner et al., 2015), the clinical utilization of expanded NIPS is still controversial (Chitty et al., 2018; Gregg et al., 2016), mainly due to its poor sensitivity, low positive prospective value, incomplete outcome data, and lack of a large patient cohort. In this study, we confirmed three cases with fetal chromosomal abnormalities other than common trisomies missed by NIPS, which had a higher frequency compared to the chance of false‐negative results with common trisomies. As test performance of whole‐genome NIPS technology on RAT and microdeletions/microduplications still need improvement, our result indicated that more clinical trials should be carried out before expended whole‐genome NIPS applied into clinical practice.

Our study highlighted the important role of ultrasound examination in NIPS cases, the four false‐negative cases in this study all showed abnormal ultrasound findings after a low‐risk NIPS result. Reiff et al. reported that unexpected ultrasound findings were seen in 3.5% of patients with a negative NIPS results during 11–14 weeks (Reiff, Little, Dobson, Wilkins‐Haug, & Bromley, 2016). In another study, Beulen et al reported on 290 patients with an abnormal fetal ultrasound who underwent NIPS, 11% of them had chromosomal abnormalities not detected by NIPS (Beulen, Faas, Feenstra, van Vugt, & Bekker, 2017). Microarray analysis revealed clinically relevant deletions or duplications in 6.0% fetuses with ultrasound anomalies and a normal karyotype (Wapner et al., 2012). Together with these previous reports, our data also demonstrated that diagnostic genetic testing using CMA should be considered in the context of abnormal ultrasound findings and a low‐risk NIPS result, consistent with the guideline “The role of ultrasound in women who undergo cell‐free DNA screening,” from Society for Maternal‐Fetal Medicine (SMFM) with the assistance of Mary E. Norton, MD; Joseph R. Biggio, MD; Jeffrey A. Kuller, MD; Sean C. Blackwell, MD. (Society for Maternal‐Fetal Medicine. Electronic address, Norton, Biggio, Kuller, & Blackwell, 2017).

A major limitation in this study is the lack of genetic diagnosis for the fetuses/neonates in 95.18% (158/166) cases with adverse pregnancy outcomes. As it was reported that 50%–60% of pregnancy losses and about 20% abnormal ultrasound findings were associated with chromosomal abnormalities (Rai & Regan, 2006; Srebniak et al., 2017; van den erg, van Maarle, van Wely, & Goddijn, 2012), the number of false‐negative results could be underestimated in this study. In addition, in most cases opted for TOP, the placental tissues were not available for any further genetic testing. Therefore, we were not able to obtain the accurate false‐negative rate as well as the underlying causes for these false negatives. The information is important for the improvement of NIPS technology, and more effort will be put in the pretest and posttest counseling in the clinical practice.

In summary, we reported four false‐negative results from following‐up 10,975 NIPS cases with a low‐risk result. Our results revealed that mosaicism contributes to a major cause of false negative in NIPS, and highlighted the importance of ultrasound in identifying these false‐negative results. In addition, our results emphasized the importance of following‐up in NIPS, especially in cases with a low‐risk results, which would cost a large number of efforts but was beneficial to realize the limitation for NIPS, and to improve prenatal management in clinic.

## CONFLICT OF INTEREST

The authors have no conflict of interest.

## References

[mgg31185-bib-0001] Benn, P. , & Grati, F. R. (2018). Genome‐wide non‐invasive prenatal screening for all cytogenetically visible imbalances. Ultrasound in Obstetrics and Gynecology, 51(4), 429–433. 10.1002/uog.19014 29363829

[mgg31185-bib-0002] Beulen, L. , Faas, B. H. W. , Feenstra, I. , van Vugt, J. M. G. , & Bekker, M. N. (2017). Clinical utility of non‐invasive prenatal testing in pregnancies with ultrasound anomalies. Ultrasound in Obstetrics and Gynecology, 49(6), 721–728. 10.1002/uog.17228 27515011 PMC5488200

[mgg31185-bib-0003] Chen, S. , Lau, T. K. , Zhang, C. , Xu, C. , Xu, Z. , Hu, P. , … Zhang, X. (2013). A method for noninvasive detection of fetal large deletions/duplications by low coverage massively parallel sequencing. Prenatal Diagnosis, 33(6), 584–590. 10.1002/pd.4110 23592436

[mgg31185-bib-0004] Cheung, K. W. , Lai, C. W. S. , Mak, C. C. Y. , Hui, P. W. , Chung, B. H. Y. , & Kan, A. S. Y. (2018). A case of prenatal isolated talipes and 22q11.2 deletion syndrome‐an important chromosomal disorder missed by noninvasive prenatal screening. Prenatal Diagnosis, 38(5), 376–378. 10.1002/pd.5241 29473648

[mgg31185-bib-0005] Chitty, L. S. , Hudgins, L. , & Norton, M. E. (2018). Current controversies in prenatal diagnosis 2: Cell‐free DNA prenatal screening should be used to identify all chromosome abnormalities. Prenatal Diagnosis, 38(3), 160–165. 10.1002/pd.5216 29417608

[mgg31185-bib-0006] Fiorentino, F. , Bono, S. , Pizzuti, F. , Duca, S. , Polverari, A. , Faieta, M. , … Spinella, F. (2017). The clinical utility of genome‐wide non invasive prenatal screening. Prenatal Diagnosis, 37(6), 593–601. 10.1002/pd.5053 28423190

[mgg31185-bib-0007] Gao, Y. , Stejskal, D. , Jiang, F. , & Wang, W. (2014). False‐negative trisomy 18 non‐invasive prenatal test result due to 48, XXX,+18 placental mosaicism. Ultrasound in Obstetrics and Gynecology, 43(4), 477–478. 10.1002/uog.13240 24186002

[mgg31185-bib-0008] Gregg, A. R. , Skotko, B. G. , Benkendorf, J. L. , Monaghan, K. G. , Bajaj, K. , Best, R. G. , … Watson, M. S. (2016). Noninvasive prenatal screening for fetal aneuploidy, 2016 update: A position statement of the American College of Medical Genetics and Genomics. Genetics in Medicine, 18(10), 1056–1065. 10.1038/gim.2016.97 27467454

[mgg31185-bib-0009] Hartwig, T. S. , Ambye, L. , Sorensen, S. , & Jorgensen, F. S. (2017). Discordant non‐invasive prenatal testing (NIPT) ‐ a systematic review. Prenatal Diagnosis, 37(6), 527–539. 10.1002/pd.5049 28382695

[mgg31185-bib-0010] Hochstenbach, R. , Page‐Christiaens, G. C. M. L. , van Oppen, A. C. C. , Lichtenbelt, K. D. , van Harssel, J. J. T. , Brouwer, T. , … Schuring‐Blom, G. H. (2015). Unexplained false negative results in noninvasive prenatal testing: Two cases involving trisomies 13 and 18. Case Reports in Genetics, 2015, 1–7. 10.1155/2015/926545 PMC4475527 26137330

[mgg31185-bib-0011] Jiang, F. , Ren, J. , Chen, F. , Zhou, Y. , Xie, J. , Dan, S. , … Zhang, X. (2012). Noninvasive Fetal Trisomy (NIFTY) test: An advanced noninvasive prenatal diagnosis methodology for fetal autosomal and sex chromosomal aneuploidies. BMC Medical Genomics, 5, 57 10.1186/1755-8794-5-57 23198897 PMC3544640

[mgg31185-bib-0012] Lefkowitz, R. B. , Tynan, J. A. , Liu, T. , Wu, Y. , Mazloom, A. R. , Almasri, E. , … Ehrich, M. (2016). Clinical validation of a noninvasive prenatal test for genomewide detection of fetal copy number variants. American Journal of Obstetrics and Gynecology, 215(2), 227.e1–227.e16. 10.1016/j.ajog.2016.02.030 26899906

[mgg31185-bib-0013] Liang, D. , Lin, Y. , Qiao, F. , Li, H. , Wang, Y. , Zhang, J. , … Xu, Z. (2018). Perinatal outcomes following cell‐free DNA screening in >32 000 women: Clinical follow‐up data from a single tertiary center. Prenatal Diagnosis, 10.1002/pd.5328 29966040

[mgg31185-bib-0014] Mennuti, M. T. , Cherry, A. M. , Morrissette, J. J. , & Dugoff, L. (2013). Is it time to sound an alarm about false‐positive cell‐free DNA testing for fetal aneuploidy? American Journal of Obstetrics and Gynecology, 209(5), 415–419. 10.1016/j.ajog.2013.03.027 23529082

[mgg31185-bib-0015] Norton, M. E. , Biggio, J. R. , Kuller, J. A. , & Blackwell, S. C. (2017). The role of ultrasound in women who undergo cell‐free DNA screening. American Journal of Obstetrics and Gynecology, 216(3), B2–B7. 10.1016/j.ajog.2017.01.005 28108156

[mgg31185-bib-0016] Pan, M. , Li, F. T. , Li, Y. , Jiang, F. M. , Li, D. Z. , Lau, T. K. , & Liao, C. (2013). Discordant results between fetal karyotyping and non‐invasive prenatal testing by maternal plasma sequencing in a case of uniparental disomy 21 due to trisomic rescue. Prenatal Diagnosis, 33(6), 598–601. 10.1002/pd.4069 23533085

[mgg31185-bib-0017] Rai, R. , & Regan, L. (2006). Recurrent miscarriage. Lancet, 368(9535), 601–611. 10.1016/S0140-6736(06)69204-0 16905025

[mgg31185-bib-0018] Reiff, E. S. , Little, S. E. , Dobson, L. , Wilkins‐Haug, L. , & Bromley, B. (2016). What is the role of the 11‐ to 14‐week ultrasound in women with negative cell‐free DNA screening for aneuploidy? Prenatal Diagnosis, 36(3), 260–265. 10.1002/pd.4774 26748490

[mgg31185-bib-0019] Schwartz, S. , Kohan, M. , Pasion, R. , Papenhausen, P. R. , & Platt, L. D. (2018). Clinical experience of laboratory follow‐up with noninvasive prenatal testing using cell‐free DNA and positive microdeletion results in 349 cases. Prenatal Diagnosis, 38(3), 210–218. 10.1002/pd.5217 29338128

[mgg31185-bib-0020] Srebniak, M. I. , Knapen, M. F. C. M. , Polak, M. , Joosten, M. , Diderich, K. E. M. , Govaerts, L. C. P. , … Van Opstal, D. (2017). The influence of SNP‐based chromosomal microarray and NIPT on the diagnostic yield in 10,000 fetuses with and without fetal ultrasound anomalies. Human Mutation, 38(7), 880–888. 10.1002/humu.23232 28409863

[mgg31185-bib-0021] van den Berg, M. M. , van Maarle, M. C. , van Wely, M. , & Goddijn, M. (2012). Genetics of early miscarriage. Biochimica Et Biophysica Acta, 1822(12), 1951–1959. 10.1016/j.bbadis.2012.07.001 22796359

[mgg31185-bib-0022] Wapner, R. J. , Babiarz, J. E. , Levy, B. , Stosic, M. , Zimmermann, B. , Sigurjonsson, S. , … Benn, P. (2015). Expanding the scope of noninvasive prenatal testing: Detection of fetal microdeletion syndromes. American Journal of Obstetrics and Gynecology, 212(3), e331–339. 10.1016/j.ajog.2014.11.041 25479548

[mgg31185-bib-0023] Wapner, R. J. , Martin, C. L. , Levy, B. , Ballif, B. C. , Eng, C. M. , Zachary, J. M. , … Jackson, L. (2012). Chromosomal microarray versus karyotyping for prenatal diagnosis. New England Journal of Medicine, 367(23), 2175–2184. 10.1056/NEJMoa1203382 23215555 PMC3549418

[mgg31185-bib-0024] Zhang, H. , Gao, Y. , Jiang, F. , Fu, M. , Yuan, Y. , Guo, Y. , … Wang, W. (2015). Non‐invasive prenatal testing for trisomies 21, 18 and 13: Clinical experience from 146,958 pregnancies. Ultrasound in Obstetrics and Gynecology, 45(5), 530–538. 10.1002/uog.14792 25598039

